# Plasticity of the Influenza Virus H5 HA Protein

**DOI:** 10.1128/mBio.03324-20

**Published:** 2021-02-09

**Authors:** Huihui Kong, David F. Burke, Tiago Jose da Silva Lopes, Kosuke Takada, Masaki Imai, Gongxun Zhong, Masato Hatta, Shufang Fan, Shiho Chiba, Derek Smith, Gabriele Neumann, Yoshihiro Kawaoka

**Affiliations:** a Influenza Research Institute, Department of Pathobiological Sciences, School of Veterinary Medicine, University of Wisconsin—Madison, Madison, Wisconsin, USA; b Department of Zoology, University of Cambridge, Cambridge, United Kingdom; c Division of Virology, Department of Microbiology and Immunology, Institute of Medical Science, University of Tokyo, Tokyo, Japan; d International Research Center for Infectious Diseases, Institute of Medical Science, University of Tokyo, Tokyo, Japan; Washington University School of Medicine

**Keywords:** HA, influenza, sequence plasticity

## Abstract

Since the emergence of highly pathogenic avian influenza viruses of the H5 subtype, the major viral antigen, hemagglutinin (HA), has undergone constant evolution, resulting in numerous genetic and antigenic (sub)clades. To explore the consequences of amino acid changes at sites that may affect the antigenicity of H5 viruses, we simultaneously mutated 17 amino acid positions of an H5 HA by using a synthetic gene library that, theoretically, encodes all combinations of the 20 amino acids at the 17 positions. All 251 mutant viruses sequenced possessed ≥13 amino acid substitutions in HA, demonstrating that the targeted sites can accommodate a substantial number of mutations. Selection with ferret sera raised against H5 viruses of different clades resulted in the isolation of 39 genotypes. Further analysis of seven variants demonstrated that they were antigenically different from the parental virus and replicated efficiently in mammalian cells. Our data demonstrate the substantial plasticity of the influenza virus H5 HA protein, which may lead to novel antigenic variants.

## Abstract (precis)

The HA protein of influenza A viruses is the major viral antigen. In this study, we simultaneously introduced mutations at 17 amino acid positions of an H5 HA expected to affect antigenicity. Viruses with ≥13 amino acid changes in HA were viable, and some had altered antigenic properties. H5 HA can therefore accommodate many mutations in regions that affect antigenicity. The substantial plasticity of H5 HA may facilitate the emergence of novel antigenic variants.

## INTRODUCTION

The antigenicity of influenza viruses is primarily determined by the viral hemagglutinin (HA) protein, in particular its head region, which harbors the major antigenic epitopes. For influenza viruses of the H1 (1, 2) and H3 (3–7) subtypes, five major antigenic epitopes each have been identified, primarily by characterizing escape mutants to mouse monoclonal antibodies directed at HA. For H5 HAs, the mapping of antigenic escape mutants onto the three-dimensional (3D) structure revealed three to five antigenic sites, depending on the virus and antibodies used for the analysis (8–12).

Highly pathogenic avian influenza viruses of the H5N1 subtype, which have caused 861 confirmed human infections since 2003 with a case fatality rate of 53% (https://www.who.int/influenza/human_animal_interface/2020_10_07_tableH5N1.pdf?ua=1), have evolved into 10 different genetic clades (0 to 9) and many subclades (13, 14). To date, relatively little is known about the antigenic evolution of highly pathogenic avian H5 influenza viruses. The limited data currently available suggest that the antigenic properties of these viruses may be determined by a small number of amino acids located around the receptor-binding site of HA (15, 16), as has been shown for human influenza viruses (17). To better understand the genetic and antigenic variability of H5 HA proteins, we simultaneously introduced mutations at 17 amino acid positions that are thought to affect the antigenicity of H5 HA. Mutant viruses with 13 or more amino acid changes in HA were viable, replicated relatively efficiently in various cell lines, and were antigenically different from wild-type (WT) virus. These data demonstrate the substantial plasticity of the HA protein, which may lead to novel antigenic variants.

## RESULTS

### Generation and characterization of an HA plasmid library encoding mutations at up to 17 amino acid positions.

**FIG 1:**
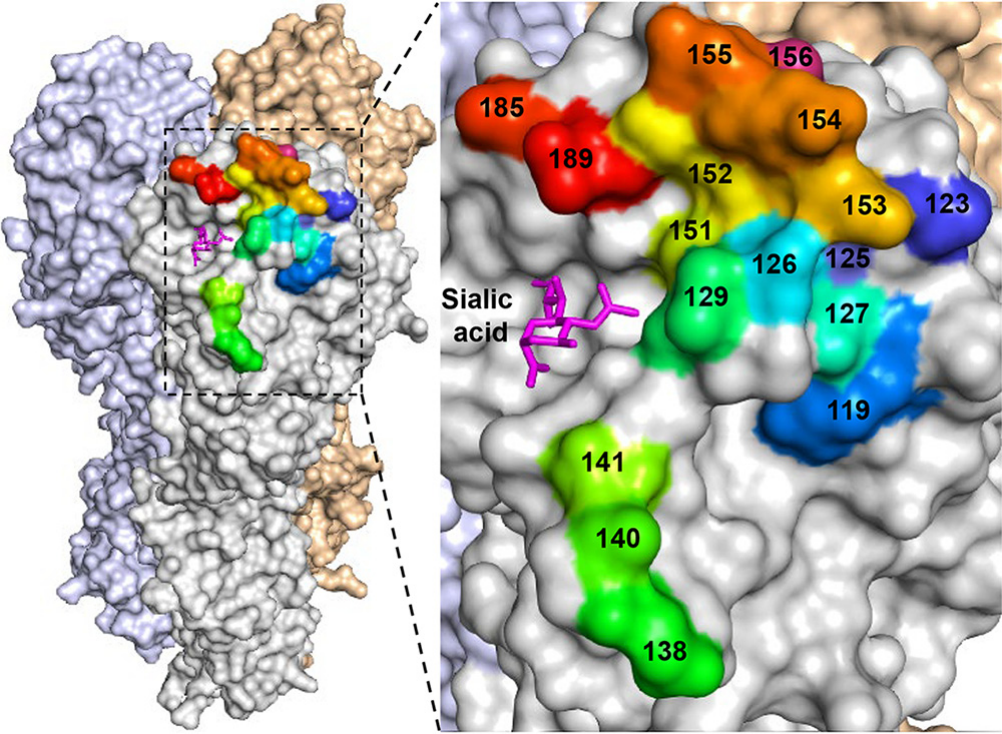
Amino acid positions selected for mutagenesis. The 3D structure of the HA trimer of Gyr virus was downloaded from the Protein Data Bank (PDB code 5HUF). The 17 amino acid positions selected for mutagenesis are indicated in different colors. Sialic acid is shown to indicate the receptor-binding site.

**FIG 2:**
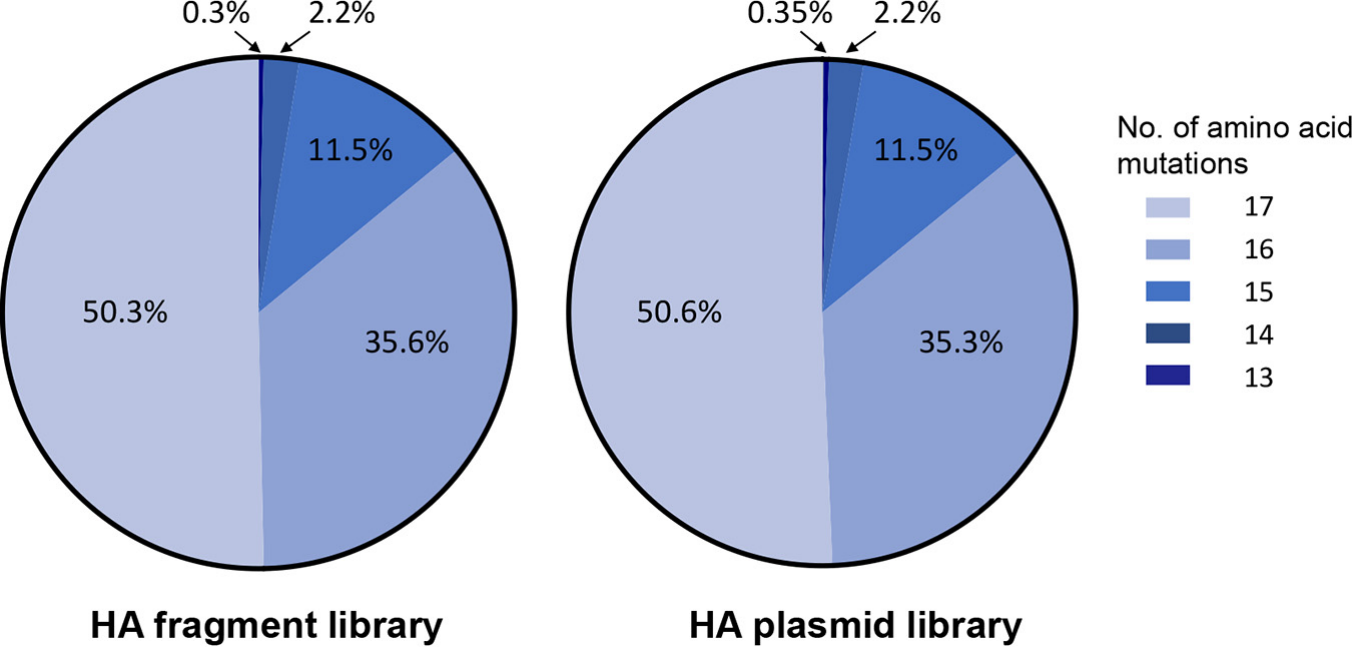
Percentages of the HA fragment and plasmid libraries encoding at least 13 amino acid changes. Shown are the percentages of next-generation sequence reads from the HA fragment and plasmid libraries encoding the indicated number of amino acid mutations.

**FIG 3:**
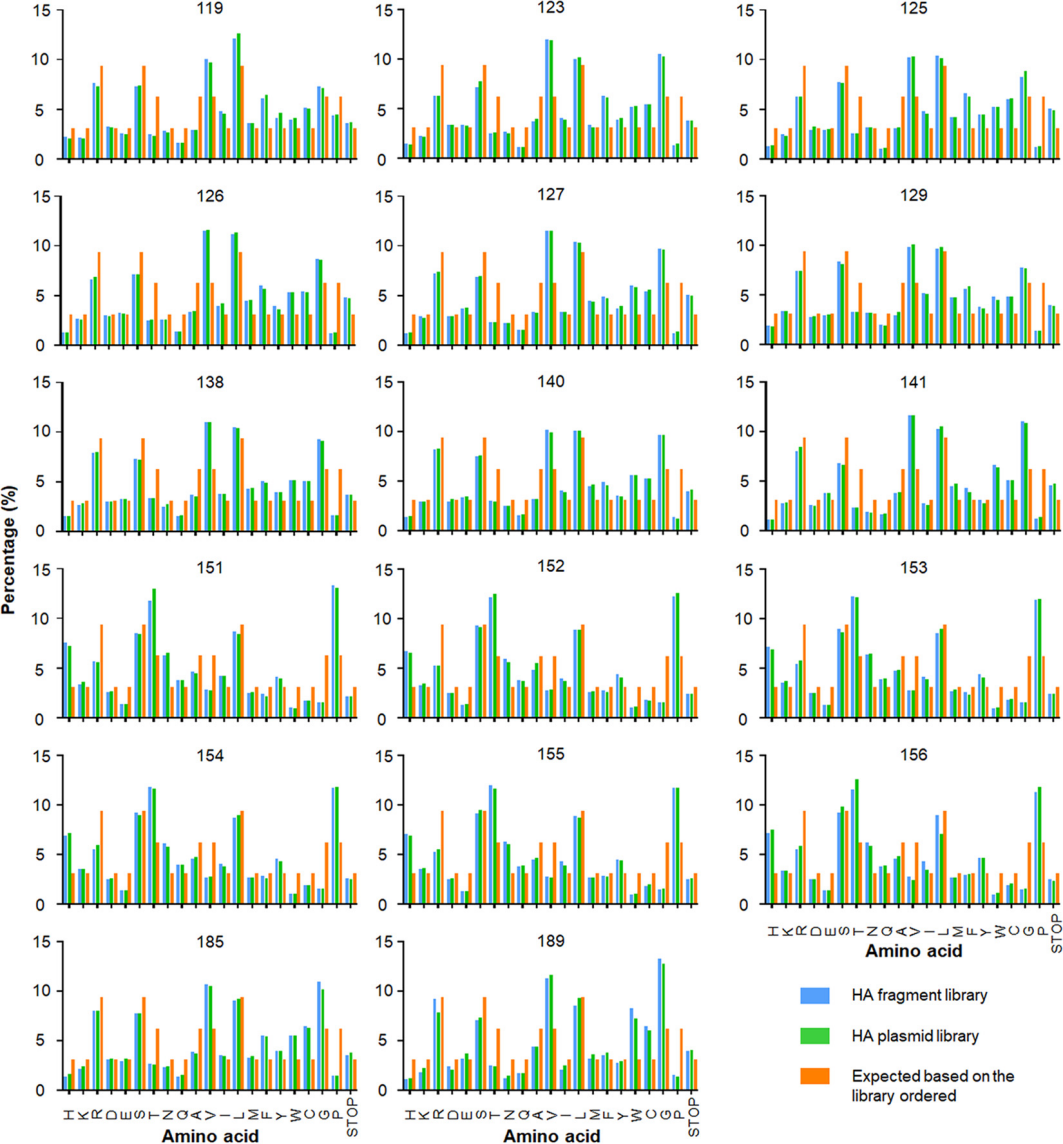
Ratios of amino acids at the 17 selected positions. The HA fragment and plasmid libraries were analyzed by using next-generation sequencing. For each of the indicated amino acid positions, the ratios of amino acids are indiciated. Blue, HA fragment library; green, HA plasmid library; orange, expected ratio based on the library ordered (see Fig. S1).

To assess the variability of H5 HA proteins, we selected a representative of the antigenic clade 2.3.4.4 of highly pathogenic avian H5 influenza viruses, namely, A/gyrfalcon/Washington/40188-6/2014 (H5N8; Gyr). This clade emerged in China around 2011-2012 and has spread to several other continents, including Europe, Africa, and North America (18–21). Based on published studies and our own data, we identified 17 amino acid positions (119, 123, 125 to 127, 129, 138, 140, 141, 151 to 156, 185 and 189; H5 numbering, used here) which are believed to affect the antigenicity of H5 HA (Table 1); these positions are located around the receptor-binding site of HA (Fig. 1). We synthesized a fragment encoding amino acids 64 to 288 of the Gyr HA protein that encodes “NNK” (N, any nucleotide; K, G or T) at the selected 17 codons. By using the NNK codon, all 20 amino acids are encoded while reducing the proportion of stop codons compared to that obtained with the “NNN” codon (see Fig. S1 in the supplemental material). The resulting HA fragment library was cloned into a plasmid vector encoding the remaining portion of HA under the control of an RNA polymerase I promoter and terminator, as previously described (22). The resulting mixture of plasmids is referred to as the HA plasmid library. The diversity of the HA fragment and HA plasmid libraries was analyzed by using next-generation sequencing. About 99% of the HA sequences from these libraries encoded mutations at at least 13 amino acid positions, with approximately 50% of the HA sequences encoding mutations at all 17 amino acid positions targeted for mutagenesis (Fig. 2): for example, 50.6% of the sequence reads from the HA plasmid library possessed mutations at all 17 targeted positions, 35.3% of the sequence reads from the HA plasmid library possessed mutations at 16 of the targeted 17 positions, etc. We also analyzed the proportions of the amino acids and found that all 20 amino acids were represented at each of the targeted positions (Fig. 3), although the amounts of some amino acids differed slightly to moderately from the ratios expected by using the NNK codon. No major differences were observed between the HA fragment and HA plasmid libraries (Fig. 3), indicating that the cloning of the synthesized HA fragment library did not result in any substantial changes in the composition of the library. Collectively, these data demonstrate the generation of an HA plasmid library with extensive sequence diversity at up to 17 amino acid positions.

**TABLE 1:** Amino acid positions selected for mutagenesis

Amino acid position		H3N2 cluster transition (17)	H1 antigenic escape (22)	H5 antigenic escape (7, 15, 16, 51)^*a*^	Antigenic epitope (7)
H3 numbering	H5 numbering				
Δ^*b*^	119			Yes	A
128	123			Yes	
130	125			Yes	B
131	126		Yes	Yes	A
132	127			Yes	A
Δ	129			Yes	A
142	138			Yes	A
144	140		Yes	Yes	A
145	141	Yes		Yes	A
155	151	Yes		Yes	B
156	152	Yes	Yes	Yes	B
157	153		Yes	Yes	B
158	154	Yes	Yes	Yes	B
159	155	Yes	Yes	Yes	B
160	156			Yes	B
189	185	Yes		Yes	B
193	189	Yes		Yes	B

a And unpublished data.

b Δ, no equivalent position in H3 HA.

10.1128/mBio.03324-20.1 FIG S1 Optimization of the amino acid ratios introduced at each position. The theoretical ratios of amino acids introduced at each position were optimized by synthesizing NNK (instead of NNN) at each amino acid position. Black, before optimization; gray, after optimization. Download FIG S1, PDF file, 0.4 MB. Copyright © 2021 Kong et al. 2021 Kong et al. This content is distributed under the terms of the Creative Commons Attribution 4.0 International license.

### Generation and characterization of a virus library encoding mutations in HA at up to 17 amino acid positions.

Previously, the Gyr virus was selected as a WHO candidate vaccine virus for H5 influenza virus (https://www.who.int/influenza/vaccines/virus/candidates_reagents/summary_a_H5_cvv_nh1920_20190220.pdf?ua=1). After the required testing, this virus was exempt from select agent regulations in the United States due to the removal of the multibasic cleavage site; therefore, it is considered a low-pathogenicity avian influenza virus (LPAIV), which the University of Wisconsin—Madison’s Institutional Biosafety Committee (IBC) has approved us to use at biosafety level 2 (BSL2). Mutant Gyr viruses encoding up to 17 amino acid changes in HA were generated by transfecting human embryonic fibroblasts (293T) with the HA plasmid library, a plasmid for the synthesis of the A/chicken/Indonesia/NC/2009 (H5N1; NC09) NA viral RNA (chosen because it confers high virus rescue efficiency), plasmids for the synthesis of the remaining six viral RNA segments of high-yield A/PR8/34 (PR8-HY) virus (23), plasmids for the synthesis of the viral polymerase and NP, and a plasmid expressing human airway trypsin-like protease, which facilitates HA cleavage (24). The supernatant derived from transfected 293T cells (i.e., the “virus library”) was collected 48 h after transfection and titrated in MDCK cells. The titer of the virus library was 2.5 × 10^4^ PFU/ml, whereas the titer of the recombinant control virus with wild-type (WT) Gyr HA was 1.1 × 10^8^ PFU/ml. The lower titer of the virus library than of the control virus encoding WT Gyr HA most likely indicated that a large percentage of viruses with mutant HAs were not viable.

To isolate individual viruses from the virus library for further characterization, we performed plaque assays in MDCK cells and sequenced the HA genes of 251 individual virus plaques. We identified 55 genotypes, all of which encoded at least 13 amino acid changes compared to WT Gyr HA (Table S1). A total of 154 viruses encoded genotype G2, which accounted for 61.3% of the viruses sequenced. Genotypes G30, G29, and G53 were detected 10, 8, and 7 times, respectively, whereas all of the other genotypes were detected no more than four times. The dominance of G2, which differs from WT Gyr HA by 15 amino acids, suggests a replicative advantage over the other variants in the virus library. Genotype G53 also encoded nucleotide insertions leading to the replacement of the amino acids KK at HA positions 152/153 with PPT; thus, this genotype differed from WT Gyr HA by a total of 18 amino acid changes. Taken together, our data show that viruses with 13 to 18 amino acid changes in the antigenic epitopes of Gyr HA are viable, demonstrating that these regions of HA can tolerate substantial sequence diversity *in vitro*.

10.1128/mBio.03324-20.2 TABLE S1 Sequence analysis of individual virus plaques isolated from the virus library. Download Table S1, XLSX file, 0.02 MB. Copyright © 2021 Kong et al. 2021 Kong et al. This content is distributed under the terms of the Creative Commons Attribution 4.0 International license.

### Isolation of antigenic escape variants.

After generating a virus library encoding mutant HA proteins, we attempted to isolate antigenic escape variants that were no longer neutralized by ferret sera raised against the parental Gyr virus, other clade 2.3.4.4 viruses, and/or viruses of clades 2.3.2.1a and 2.3.2.1b (selected because viruses of these clades were prevalent before the emergence of clade 2.3.4.4 [25, 26]). Specifically, we incubated the virus library with the ferret antisera indicated in Table S2 and subjected the virus/serum mixtures to plaque assays in MDCK cells. We isolated 93 viruses representing 39 different genotypes (Table S2). Genotype G2, which was dominant in the virus library, was no longer detected, suggesting that it was neutralized by the ferret antisera, even though its HA differed by multiple amino acids from those of the viruses used for serum generation. Rather, genotype G30 became the most frequently detected genotype after antigenic selection, being detected in 25 of 93 virus plaques (26.9%); it differs by 17 amino acids from Gyr HA. Genotypes identified more than once were typically selected with several ferret antisera (including those raised against viruses of clades 2.3.2.1a and 2.3.2.1b) (Table S2), indicating that the selected variants were substantially different from clade 2.3.4.4 and clade 2.3.2.1 viruses. Most of the genotypes isolated after antigenic selection had not been detected among the 251 virus plaques characterized from the virus library, probably because the sequence analysis of 251 virus plaques does not capture the full genetic diversity of the virus library.

10.1128/mBio.03324-20.3 TABLE S2 Sequence analysis of antigenic escape mutants. Download Table S2, XLSX file, 0.02 MB. Copyright © 2021 Kong et al. 2021 Kong et al. This content is distributed under the terms of the Creative Commons Attribution 4.0 International license.

### Characterization of antigenic escape variants.

**FIG 4:**
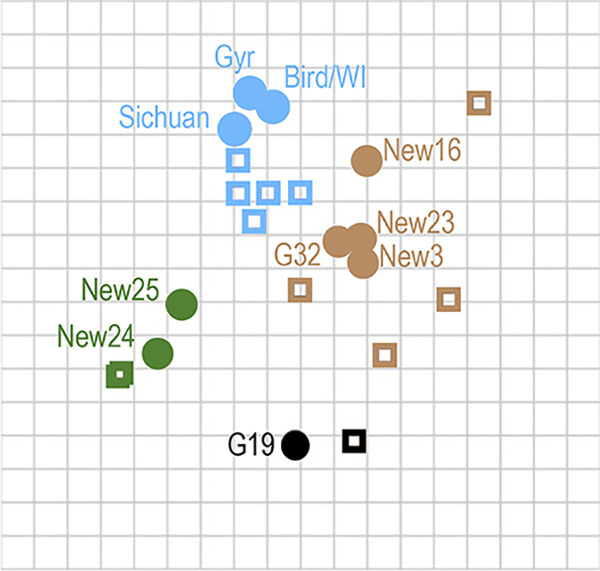
Antigenic cartography of the mutants. An antigenic map was generated by using the HI assay data shown in Table 2. Each square represents a 2-fold difference in HI titers. Viruses and sera are represented by circles and rectangles, respectively. Clade 2.3.4.4 wild-type viruses are shown in blue (Gyr, A/gyrfalcon/Washington/41088-6/2014_PR8-HY; Sichuan, A/Sichuan/26221/2014; Bird/WI, A/bird/Wisconsin/WDSL1-4/2015). Gyr mutants are shown in brown (antigenic group 1), green (antigenic group 2), and black (antigenic group 3).

The isolated Gyr mutants bear 13 to 18 amino acid changes at positions thought to affect antigenic properties. To test whether these mutants were antigenically different from WT Gyr, we tested 35 of the 39 genotypes isolated after antigenic selection, together with the dominant genotype before antigenic selection (G2), against ferret sera to WT Gyr and A/Sichuan/26221/2014, another clade 2.3.4.4 virus. The remaining four genotypes isolated after antigenic selection (i.e., G12, G17, New2, and New6) were excluded from further characterization because they did not replicate efficiently. Hemagglutination inhibition (HI) assays demonstrated that many mutants lost their reactivity with sera raised against the clade 2.3.4.4 viruses (Table S3). To further characterize the antigenicity of selected Gyr mutants (i.e., mutants G19, G32, New3, New16, New23, New24, and New25; shown in boldface in Table S3), we raised ferret antisera against recombinant viruses encoding the mutant Gyr HA in combination with Gyr neuraminidase (NA) and the remaining viral RNA segments of PR8-HY. HI assays were then carried out to test the reactivity of the sera against mutant and wild-type Gyr viruses (Table 2). The HI data were used for antigenic cartography (27), in which the relative HI distances among viruses are presented in two- or three-dimensional antigenic maps (Fig. 4). In these antigenic maps, two grid units represent a 4-fold difference in HI titers, which may necessitate a vaccine update. As shown in Fig. 4, the three clade 2.3.4.4 wild-type viruses (blue circles) are located close to each other and to ferret sera raised against them (blue squares), demonstrating the robustness of the antigenic map. All seven Gyr mutants were antigenically different from the WT Gyr virus, demonstrating that the amino acid changes in HA affected the antigenicity of the viruses. Four Gyr mutants (G32, New3, New16, and New23) formed antigenic group 1 (Fig. 4, brown), which was located closer to the wild-type viruses than the other three Gyr mutants. Viruses in antigenic group 2 (New24 and New25; Fig. 4, green) and antigenic group 3 (G19; Fig. 4, black) localized to antigenic spaces at an appreciable distance from the clade 2.3.4.4 wild-type viruses. These data demonstrate that our experimental approach of mutating 17 amino acid positions at once yielded replicating viruses with novel antigenic properties.

**TABLE 2:** HI titers of selected mutant Gyr viruses against sera raised to homologous and heterologous viruses^*a*^

Virus	Clade	Ferret serum											
		A/Sichuan/26221/2014 (IDCDC-RG42A) (H5N6)_F24_15	A/Sichuan/26221/2014 (IDCDC-RG42A) (H5N6)_F23_15	A/bird/Wisconsin/WDSL1-4/2015_5974	A/bird/Wisconsin/WDSL1-4/2015_5975	A/gyrfalcon/Washington/41088-6/2014_PR8-HY_5973	G19_2968	New3_2966	New16_3641	G32_3644	New23_3648	New24_5388	New25_4399
A/bird/Wisconsin/WDSL1-4/2015	2.3.4.4	<28	14	**372**	**226**	141	<10	<10	<10	<10	<10	<10	<10
A/Sichuan/26221/2014 (IDCDC-RG42A)	2.3.4.4	**194**	**160**	10	33	320	<10	<10	<10	<10	<10	<10	<10
A/gyrfalcon/Washington/41088-6/2014_PR8-HY	2.3.4.4	20	<10	181	33	**452**	<10	<10	<10	<10	<10	<10	<10
G19	Antigenic group 3	<10	<10	<10	<10	47	**2,560**	226	<10	<10	<10	80	40
New3	Antigenic group 1	14	10	80	80	113	80	**2,560**	<10	<10	160	<10	20
New16	Antigenic group 1	28	14	80	80	80	40	40	**905**	10	28	<10	10
G32	Antigenic group 1	10	56	40	113	80	20	160	10	**320**	160	<10	20
New23	Antigenic group 1	28	20	80	80	80	28	160	20	56	**2,560**	<10	40
New24	Antigenic group 2	<10	10	30	<10	<10	20	40	<10	10	<10	**2,560**	2,560
New25	Antigenic group 2	30	40	60	28	25	10	20	<10	10	80	2,560	**5,120**

a Shown are the averages of 2 to 12 HI assays. Homologous titers are shown in boldface.

10.1128/mBio.03324-20.4 TABLE S3 Antigenic characterization of the escape mutants. Download Table S3, XLSX file, 0.01 MB. Copyright © 2021 Kong et al. 2021 Kong et al. This content is distributed under the terms of the Creative Commons Attribution 4.0 International license.

### Growth kinetics of mutant Gyr viruses.

**FIG 5:**
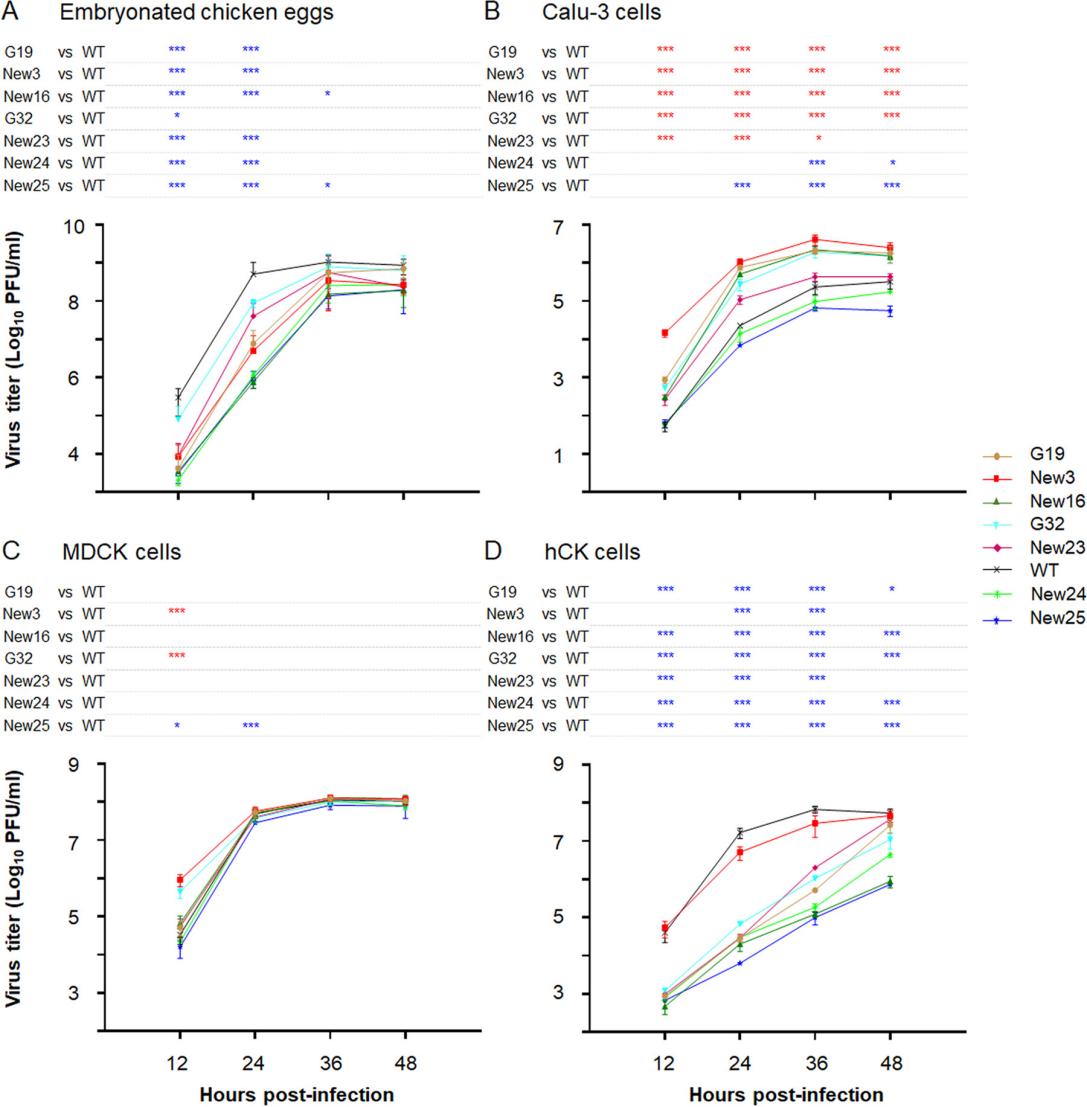
Growth kinetics of mutant Gyr viruses. The growth kinetics of the mutant Gyr viruses (with Gyr NA and the remaining genes from PR8-HY) were evaluated in embryonated chicken eggs (A), Calu-3 cells (B), MDCK cells (C), and hCK cells (D). The embryonated chicken eggs were infected with 1 × 10^3^ PFU of the indicated viruses and incubated at 37°C. Calu-3 cells were infected at an MOI of 0.03 and incubated at 33°C, whereas MDCK and hCK cells were infected at an MOI of 0.001 and incubated at 37°C. The cell supernatants were collected at the indicated time points and titrated in MDCK cells. The data shown are the averages ± SDs from three independent experiments. Red asterisks, titer is significantly higher than that of WT virus; blue asterisks, titer is significantly lower than that of WT virus; *, *P < *0.05; **, *P < *0.01; ***, *P < *0.001.

Next, we evaluated the growth kinetics of the WT and mutant Gyr viruses (possessing the WT or mutant Gyr HA genes, the Gyr NA gene, and the remaining genes derived from PR8-HY) in embryonated chicken eggs and different mammalian cell lines (Fig. 5). In embryonated chicken eggs (Fig. 5A), most of the mutant Gyr viruses replicated less efficiently than the WT Gyr virus at 12 h and 24 h postinfection (p.i.). At 36 h and 48 h p.i., several mutants replicated to titers similar to those of the WT Gyr virus.

In human bronchial epithelial Calu-3 cells (Fig. 5B), the relative growth kinetics of the WT and mutant Gyr viruses were different from those in embryonated chicken eggs. Specifically, five mutant Gyr viruses (G19, G32, New3, New16, and New23) replicated more efficiently than the WT Gyr virus at several time points. The New24 and New25 mutants were mildly to moderately attenuated compared to the WT Gyr virus. We also assessed the viral growth kinetics in MDCK cells and humanized MDCK (hCK) cells (28), which express reduced levels of α2,3-linked sialic acids (the preferred receptor of avian influenza viruses) and increased levels of α2,6-linked sialic acids (the preferred receptor of human influenza viruses). In MDCK cells (Fig. 5C), the WT and mutant Gyr viruses displayed similar growth kinetics at most time points, although two mutants (New3 and G32) replicated more efficiently than the WT Gyr virus at 12 h p.i. In hCK cells (Fig. 5D), the mutant Gyr viruses were strongly attenuated compared to the WT Gyr virus at most time points, with the exception of the New3 virus, which was mildly attenuated. Collectively, these data demonstrate that the mutant viruses replicated efficiently in mammalian cells, despite the appreciable number of amino acid changes in their HA proteins.

### Hemadsorption activity of selected mutants.

**FIG 6:**
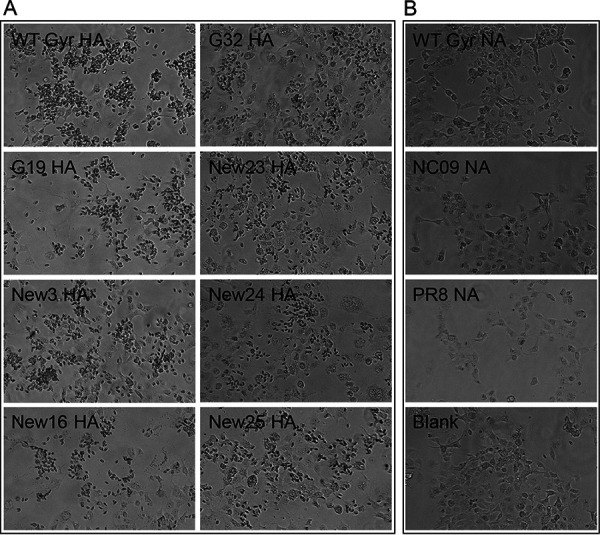
Hemadsorption assay. The hemadsorption assay was performed with TRBCs in COS-1 cells expressing HA (A) or NA (B) protein. HA- or NA-expressing cells were incubated with TRBCs at 48 h posttransfection for 30 min at room temperature, washed, and observed under a light microscope.

The 17 amino acid positions targeted for mutagenesis cluster around the receptor-binding site of HA, and mutations at these positions may affect the hemadsorption and receptor-binding activity of HA (29). We therefore performed hemadsorption assays by expressing WT or mutant Gyr HA proteins in African green monkey kidney fibroblasts (COS-1). The HA-expressing cells were incubated with turkey red blood cells (TRBCs), and TRBC binding to HA-expressing cells was assessed by use of light microscopy. All mutant HA proteins bound to TRBCs to various degrees (Fig. 6A). Because the NA protein can also exhibit hemadsorption activity (30), which could compensate for reduced mutant HA hemadsorption activity (31, 32), we assessed the potential contribution of NA to virus attachment by also expressing the NA proteins of NC09, Gyr, and PR8 viruses in COS-1 cells. Under these conditions, the NA proteins showed no hemadsorption activity (Fig. 6B). Collectively, our data confirm that the multiple amino acid substitutions in HA do not abrogate its hemagglutination activity and that the NA proteins tested do not affect the hemagglutination activity measured in our study.

### Receptor binding preference of mutant Gyr viruses.

**FIG 7:**
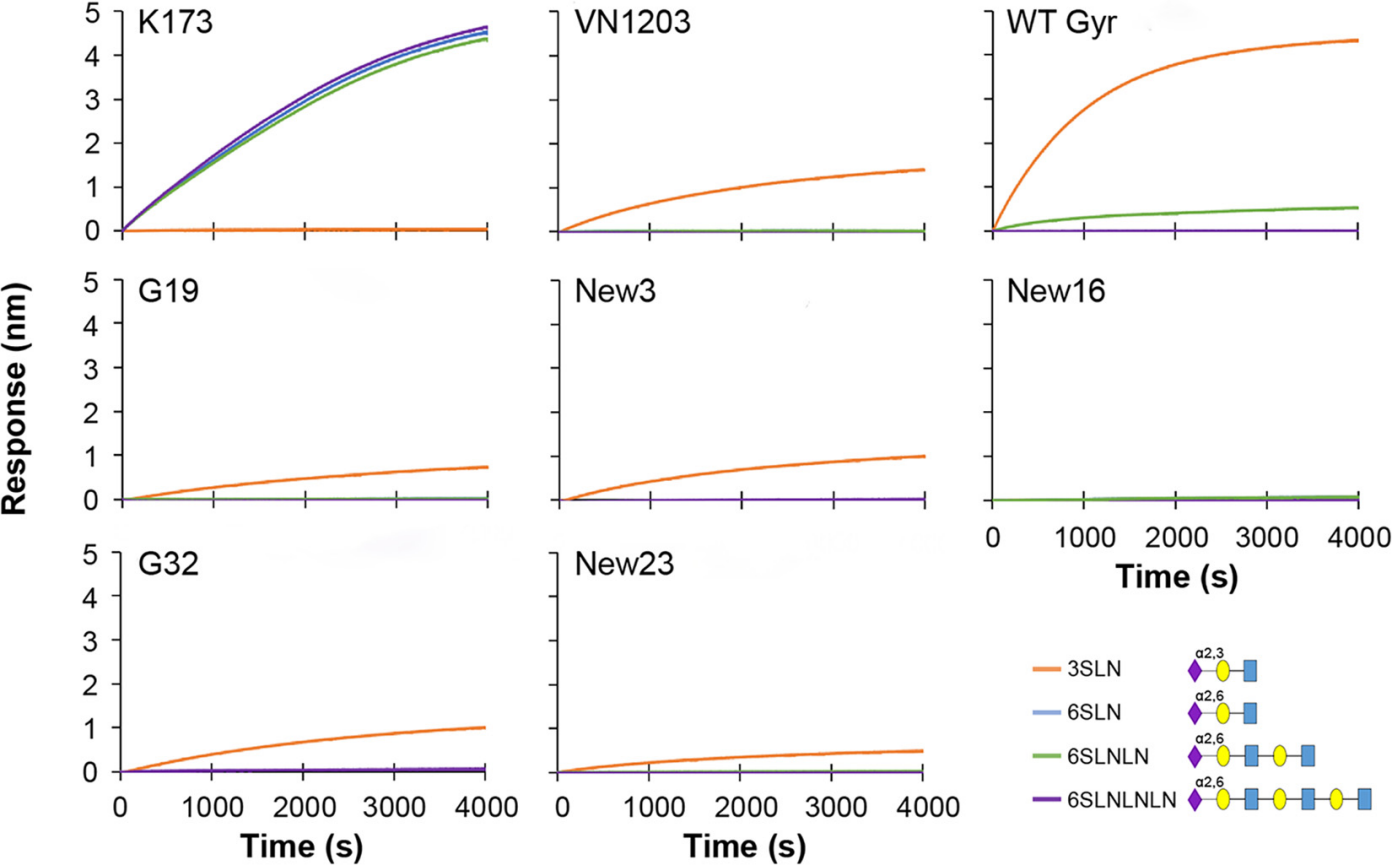
Receptor binding preferences of mutant Gyr viruses. The receptor binding preferences of mutant Gyr viruses (with Gyr NA and the remaining genes from PR8-HY) were evaluated by using biolayer interferometry. The α2,3-linked (3SLN) and α2,6-linked glycans (6SLN, 6SLNLN, and 6SLNLNLN) were immobilized to streptavidin biosensors and reacted with purified viruses in the presence of 10 mM oseltamivir carboxylate. Association was measured for 4,000 s at 30°C. Blue squares, *N*-acetylglucosamine; yellow circles, galactose; purple diamonds, sialic acid.

To study the receptor binding preference of selected mutant Gyr viruses in more detail, we performed surface biolayer interferometry with purified G19, New3, New16, G32, and New23 viruses, which replicated more efficiently than the WT Gyr virus in Calu-3 cells (Fig. 5). A/Kawasaki/173/2001 (H1N1; K173), a human influenza virus that preferentially binds α2,6-linked glycans, and A/Vietnam/1203/2004 (H5N1; VN1203), which binds preferentially to α2,3-linked glycan, served as controls. The WT Gyr virus bound to the α2,3-linked glycan with high efficiency; in addition, low binding activity to one of the α2,6-linked glycans was detected (Fig. 7). Compared with the WT Gyr virus, several mutants showed decreased binding activity to the α2,3-linked glycan; however, their binding activity to α2,3-linked glycan was similar to that of VN1203 (Fig. 7). For mutant New16, no substantial binding to the α2,3-linked glycan tested was detected. None of the mutant Gyr viruses displayed appreciable binding to the α2,6-linked glycans. Thus, most of the mutant Gyr viruses maintained some ability to bind to α2,3-linked glycans and did not acquire substantial ability to bind to α2,6-linked glycans.

## DISCUSSION

Here, we demonstrated that H5 viruses with 13 to 18 simultaneously introduced amino acid substitutions in their HA antigenic epitopes are viable, replicate relatively efficiently in mammalian cells, and differ antigenically from the wild-type virus.

Currently, 10 major clades of H5 viruses are recognized, most of them with multiple subclades. The current clade designation is based on the viral HA sequence rather than on the antigenic relationships of these viruses. However, the genetically defined (sub)clades typically also differ antigenically (15, 33–35), demonstrating that (some of) the sequence differences among the (sub)clades also affect antigenicity. Currently, it is not known how many additional (sub)clades of H5 viruses may emerge. Here, we demonstrate that by mutating HA amino acid positions known or predicted to affect antigenicity, viruses can be generated whose antigenic properties greatly differ from those of the parent virus. Since our study tested only a small proportion of the theoretically possible sequence variants, it is highly likely that many more Gyr variants would be viable and antigenically different from the WT Gyr virus.

In the antigenic map, the seven Gyr mutants tested formed three antigenic groups away from the wild-type clade 2.3.4.4 viruses. Sequence analysis revealed that the four viruses in antigenic group 1 share no more than 3 of the 17 mutated amino acids, whereas the two viruses in antigenic group 2 share no more than 5 of the 17 mutated amino acids. These findings draw attention to our limited understanding of the correlation between primary amino acid sequence and antigenic properties. However, with the experimental approaches that are now in place (reference 22 and this study), large numbers of mutants can be generated and screened for desired antigenic properties, such as escape from immunity against currently circulating viruses. The ability to generate viruses with novel antigenic properties could open the door to develop candidate vaccine viruses that provide protection against viruses of various antigenic clusters, against antigenic clusters that may emerge in the future, or both.

The simultaneous mutagenesis at 17 amino acid positions would be expected to result in a high percentage of HA genes encoding stop codons or amino acids that do not yield viable virus. In addition, epistatic effects, where the amino acid at one position affects the amino acid at another position, would be expected to limit the number of genotypes resulting in replication-competent viruses. Nonetheless, our analysis of 251 virus plaques revealed 55 genotypes of highly diverse sequences at the mutated amino acid positions. In fact, at each of the mutated positions, we detected 12 to 18 different amino acids representing different biophysical categories (Table S1). The most dominant genotype (G2) differed by 15 amino acids from WT Gyr HA; most of these amino acid substitutions changed the biophysical properties of the residue at the respective position. Sequence diversity has been reported at many amino acid positions of influenza virus proteins. In particular, antigenic sites including amino acid positions close to the receptor-binding region have an appreciable tolerance to mutation (36–41), but our analysis revealed greater than previously appreciated sequence flexibility across multiple amino acid positions and antigenic epitopes.

After antigenic selection with several ferret antisera to different H5 virus clades, we identified 39 different genotypes. We still observed considerable genetic diversity, but the number of different amino acids per position was slightly reduced compared to the virus library before antigenic selection, indicating a narrower range of amino acids that conferred antigenic escape.

An important finding of our study is that viruses with 13 to 18 simultaneously introduced mutations in HA are replication competent and, dependent on the cell type, may even replicate more efficiently than the parental virus. However, such variants are unlikely to emerge in nature because of the large number of mutations that would need to occur simultaneously. In fact, our analysis of all of the H5 HA sequences available in GenBank did not identify HAs identical to the mutants characterized here. However, at individual positions, the isolated mutants possess amino acids that have been detected among circulating H5 viruses (Table S4). For example, genotype G2 encodes leucine at positions 123 and 125, which have both been detected in nature, albeit not necessarily in the same virus. Collectively, our findings highlight the sequence and antigenic flexibility of influenza virus HA proteins, which may give rise to influenza viruses with altered antigenic properties. Such variants may open the door for new vaccine design.

10.1128/mBio.03324-20.5 TABLE S4 Amino acid analysis of individual virus plaques isolated from the virus library. Download Table S4, XLSX file, 0.02 MB. Copyright © 2021 Kong et al. 2021 Kong et al. This content is distributed under the terms of the Creative Commons Attribution 4.0 International license.

## MATERIALS AND METHODS

### Viruses and cells.

293T human embryonic cells and COS-1 African green monkey kidney fibroblasts (ATCC, USA) were maintained in Dulbecco’s modified Eagle medium (DMEM) containing 10% fetal bovine serum (FBS). Polarized human bronchial epithelial Calu-3 cells (ATCC) were maintained in DMEM/nutrient mixture F-12 containing 10% FBS. MDCK and hCK (28) cells were grown in MEM supplemented with 5% newborn calf serum and maintained in MEM containing 0.3% bovine serum albumin (BSA). The HA gene of the highly pathogenic clade 2.3.4.4 virus A/gyrfalcon/Washington/40188-6/2014 (H5N8) was modified to encode a single basic amino acid at the HA cleavage site. The modified HA viral RNA segment was combined with the viral PB2, PB1, PA, NP, M, and NS viral RNA segments of a high-yield version of A/Puerto Rico/8/34 (H1N1) virus (23) and the NA viral RNA segments of Gyr, NC09, or PR8, resulting in viruses that were exempt from select agent regulations and could be used under biosafety level 2 (BSL2) containment.

### Gene mutagenesis and construction of a plasmid library.

Mutagenensis of the HA gene was achieved by synthesizing a DNA fragment (Blue Heron Biotech, USA) that spans amino acid positions 64 to 288 of the A/gyrfalcon/Washington/40188-6/2014 HA. At each of the 17 selected amino acid positions, the codon NNK allowed the synthesis of all 20 amino acids while reducing the percentage of stop codons compared to that with NNN (see Fig. S1). The synthesized gene fragment was amplified by PCR using Phusion high-fidelity DNA polymerase (Thermo Fisher, USA). The plasmid library was constructed by replacing the corresponding region of an RNA polymerase I plasmid encoding A/gyrfalcon/Washington/40188-6/2014 viral HA (with a single basic amino acid at the HA cleavage site) with the PCR product of the synthesized DNA fragment as previously reported (22). The quality of the synthesized HA fragment and HA plasmid libraries was evaluated by performing next-generation sequencing.

### Virus library generation.

To generate a virus library, we cotransfected the HA plasmid library into 293T cells with seven RNA polymerase I-controlled plasmids synthesizing the NA gene of NC09 and the six internal genes of PR8-HY, four plasmids expressing the viral replication complex (PB2, PB1, PA, and NP), and one plasmid expressing human airway trypsin-like protease, which facilitates HA cleavage, by using the method described by Neumann et al. (42). The transfection was carried out with TransIT-293 reagent (Mirus, USA). Forty-eight hours posttransfection, the 293T cell supernatant was collected for subsequent use.

### Hemagglutination and HI assays.

Hemagglutination assays were performed by adding 50 μl of 0.5% TRBCs to 50 μl of 2-fold serial dilutions of viruses in 96-well V-bottom plates. The plates were incubated at room temperature for 30 min. The hemagglutination titer is the highest dilution that agglutinates the TRBCs.

HI assays were conducted to evaluate the highest dilution of serum that prevents hemagglutination. The serum was treated with receptor-destroying enzyme (RDE; Denka Seiken, Japan) as described elsewhere (43). Then, 25 μl of 2-fold serial dilutions of RDE-treated sera were mixed for 30 min at room temperature with 25 μl of virus containing 4 HA units. Finally, 50 μl of 0.5% TRBCs was added to the serum, and the mixtures were incubated for another 45 min at room temperature. The HI titer is the highest serum dilution that inhibits TRBC agglutination.

### Hemadsorption assay.

The HA or NA proteins of the desired viruses were expressed in COS-1 cells by transfecting the cells with pCAGGS-HA or -NA protein expression vectors using Lipofectamine 2000 (Thermo Fisher Scientific, USA). Twenty-four hours later, the transfection medium was replaced with DMEM containing 10% FBS, and the cells were incubated for another 24 h. To assess the hemadsorption activity, the cells were washed with Opti-MEM twice and then incubated with 500 μl of 0.5% TRBCs at room temperature for 30 min. After being washed five times with Opti-MEM, the cells were observed under a light microscope (Thermo Fisher Scientific, USA).

### Antigenic cartography of saturated mutants.

The antigenicity distances among viruses were assessed as described previously (27). The antigenic distance among viruses was based on the HI titers of different sera tested against them, calculated using the equation *D_ij_* = *b_j_* − log_2_ (*H_ij_*), where *D_ij_* is the target distance between virus *i* and serum *j*, *H_ij_* is the titer of virus *i* against serum *j*, and *b_j_* is log_2_ value of the highest titer against serum *j*. The error function was minimized using multiple random restarts of the conjugant gradient optimization method.

### Evaluation of viral growth efficiency.

To analyze viral growth kinetics in eggs, 10^3^ PFU of viruses were injected into 10-day-old embryonated eggs in quadruplicate. The eggs were incubated at 35°C and allantoic fluids were collected at 12-h intervals. MDCK and hCK cells were infected at a multiplicity of infection (MOI) of 0.001, whereas Calu-3 cells were infected at an MOI of 0.03; all infections were carried out in triplicate. Briefly, the cells were washed with phosphate-buffered saline (PBS) twice, infected with the desired MOI of viruses diluted in MEM/BSA, incubated for 1 h, and again washed twice. The cells were maintained in MEM/BSA containing 0.5 μg/ml of tosyl phenylalanyl chloromethyl ketone trypsin at 37°C for MDCK and hCK cells and at 33°C for Calu-3 cells. Aliquots of cell supernatants were collected at 12-h intervals. The titers of the collected allantoic fluids and cell supernatants were titrated in MDCK cells by performing plaque assays.

### Generation of ferret antisera.

Ferret antisera were generated by using 6- to-10-month-old female ferrets (Triple F Farms, USA) that were serologically negative for circulating seasonal influenza A and B viruses. For each virus, two ferrets were intranasally inoculated with 10^6^ PFU of the desired virus in a volume of 500 μl (250 μl per nostril). The ferrets were boosted with 100 μg of purified virus 3 weeks after infection. The sera were collected by euthanizing the ferrets 3 weeks after the boost. All ferret experiments were performed by following the Animal Care and Use Committee guidelines of the University of Wisconsin—Madison (protocol number V00806).

### Sequence analysis.

To analyze the quality of the HA fragment and plasmid libraries, the fragment and plasmid libraries were analyzed by means of next-generation sequencing. The mutated region of HA was PCR amplified with oligonucleotides possessing library adapter sequences. The amplified samples were processed and sequenced by the University of Wisconsin—Madison Biotechnology Center on a MiSeq platform using the MiSeq reagent kit v3 (600 cycle) (Illumina, USA).

The next-generation sequencing data were analyzed by aligning the paired-end reads of each sample (libraries or control) to the Gyr HA reference sequence, using BWA mem (44). Next, the resulting BAM files were sorted and indexed using SAMTools (45). We then developed custom in-house scripts using two R statistical packages (https://www.r-project.org/), BioStrings (46) and GenomicAlignments (47), to obtain a consensus between the paired-reads and translated them from nucleotides to amino acids. Finally, for each sample separately, our script selected and concatenated the amino acids at the selected 17 positions described here (namely, 119, 123, 125, 126, 127, 129, 138, 140, 141, 151, 152, 153, 154, 155, 156, 185, and 189), counted the number of times each motif was detected in the entire data set, and calculated the percentages.

Individual viral gene segments were sequenced by using Sanger sequencing. Briefly, viral RNA was extracted from the virus supernatant or purified plaques by using the QIAmp viral RNA minikit (Qiagen, USA). The PCR product was amplified by using the SuperScript III one-step reverse transcription-PCR (RT-PCR) system with Platinum *Taq* DNA polymerase (Thermo Fisher Scientific, USA). The samples were sequenced on an Applied Biosystems DNA analyzer at the Biotechnology Center of the University of Wisconsin—Madison, and the data were analyzed by using DNASTAR software.

### Biolayer interferometry.

The receptor binding preference was analyzed by using the Octet Red 96 system (FortéBio, USA) in 96-well microplates as previously described (48). Four types of biotinylated glycans, Neu5Ac(α2-3)Gal(β1-4)GlcNAc (3SLN), Neu5Ac(α2-6)Gal(β1-4)GlcNAc (6SLN), Neu5Ac(α2-6)Gal(β1-4)GlcNAc(β1-3)Gal(β1-4)GlcNAc (6SLNLN), and Neu5Ac(α2-6)Gal(β1-4)GlcNAc(β1-3)Gal(β1-4)GlcNAc(β1-3)Gal(β1-4)GlcNAc (6SLNLNLN), all coupled with polyglutamic acid-biotin, were provided by Tokyo Chemical Industry. Viruses purified through 30% sucrose in PBS were used in this analysis. First, the streptavidin biosensors (FortéBio) were immobilized with 1 mg/ml of sialoglycopolymers in 150 mM NaCl, 10 mM HEPES (pH 7.4), 3 mM EDTA, and 0.005% surfactant P20 (HBS-EP). Then the biosensors were reacted with virus samples containing 128 HA units in HBS-EP containing 10 mM oseltamivir carboxylate. The reaction ran for 4,000 s at 30°C. Octet data analysis software was used to analyze the association data.

### Statistical analysis.

For the analysis of the growth curve data from cell lines, we performed a linear mixed-effects analysis. As fixed effects, we used the viruses and the time of the measurement (with an interaction term between those fixed effects). As random effects, we had intercepts for the individual replicates. We also transformed the virus titer values to the log 10 scale and used the R statistical package (https://www.r-project.org/), lme4 (49), and the EMMeans package (50) for the group comparisons. The *P* values were adjusted using Holm’s method.

To analyze the viral growth curves in embryonated chicken eggs, we used a two-way analysis of variance (ANOVA), followed by Dunnett’s test to compare the mutants with the wild type.

In all analyses, we considered the differences significant if the *P* values were less than 0.05.

### Biosafety statement.

All recombinant DNA protocols were approved by the University of Wisconsin—Madison’s Institutional Biosafety Committee (IBC) after risk assessments were conducted by the Office of Biological Safety. All experiments were approved by the University of Wisconsin—Madison’s IBC. The manuscript was reviewed by the University of Wisconsin—Madison Dual Use Research of Concern (DURC) Subcommittee. This review was conducted in accordance with the U.S. Government September 2014 DURC policy. The DURC Subcommittee concluded that the studies described here meet the criteria of dual use research (DUR), but not those of DURC. In addition, the University of Wisconsin—Madison Biosecurity Task Force regularly reviews the research, policies, and practices of research conducted with pathogens of high consequence at the institution. This task force has a diverse skill set and provides support in the areas of biosafety, facilities, compliance, security, law, and health. Members of the Biosecurity Task Force are in frequent contact with the principal investigator and laboratory personnel to provide oversight and ensure biosecurity.
