# Supplementary figures and images for: Plasticity of the Influenza Virus H5 HA Protein
Source: mBio. 2021 Feb 9;12(1):e03324-20. doi: 10.1128/mBio.03324-20 (PMC7885105; doi:10.1128/mBio.03324-20)

Figure S1

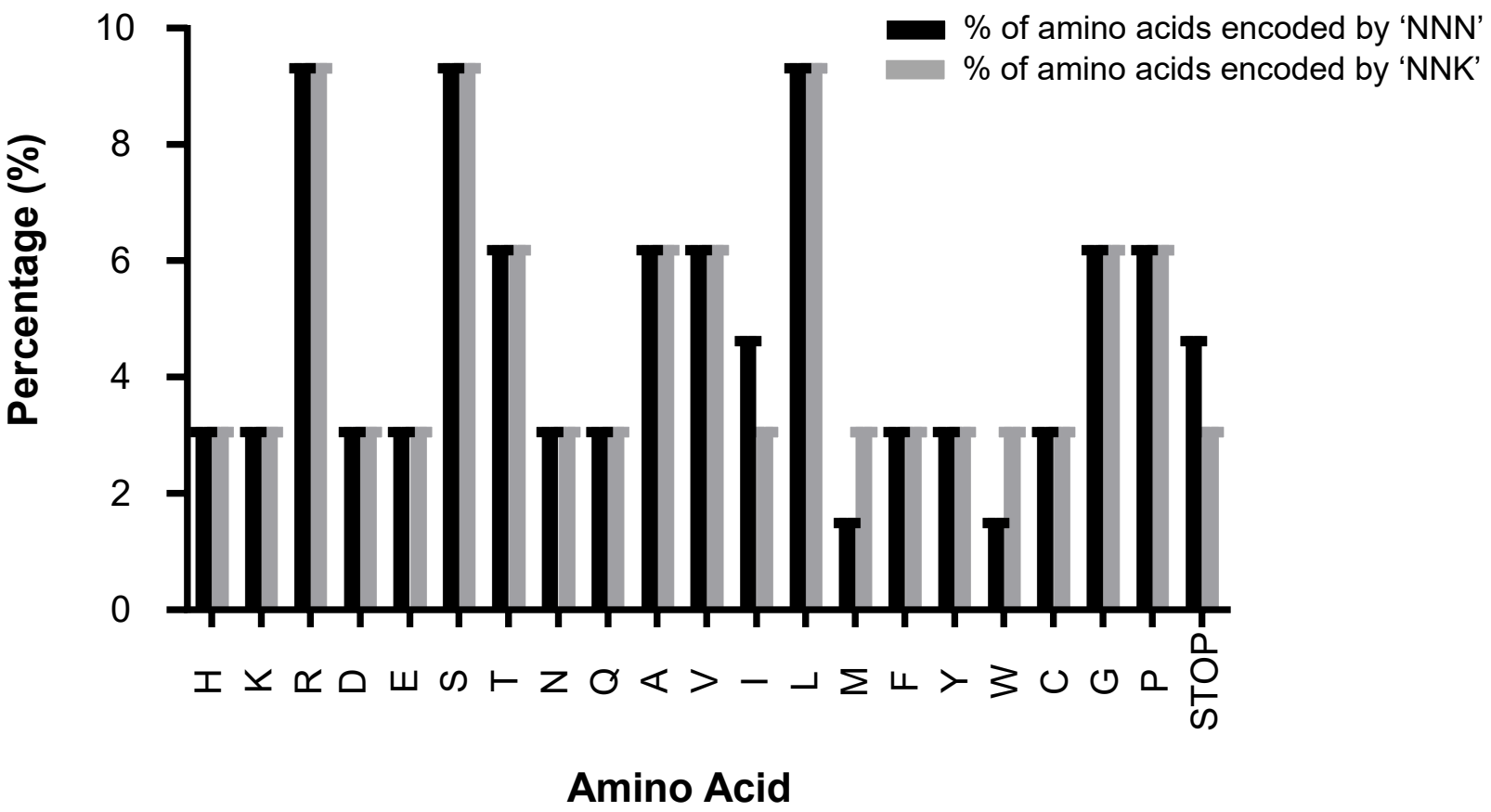

Supplement: FIG S1 [file mBio.03324-20-sf001.pdf]
